# Adaptive Deep Brain Stimulation for sleep stage targeting in Parkinson’s disease

**DOI:** 10.1016/j.brs.2023.08.006

**Published:** 2023-08-09

**Authors:** Clay Smyth, Md Fahim Anjum, Shravanan Ravi, Timothy Denison, Philip Starr, Simon Little

**Affiliations:** a Department of Bioengineering, University of California, San Francisco, UCSF Byers Hall Box 2520, 1700 Fourth St Ste 203, San Francisco, CA, 94143, United States; b Department of Neurology, University of California, San Francisco, Eighth Floor, 400 Parnassus Ave, San Francisco, CA, 94143, United States; c Department of Engineering Science, University of Oxford, Parks Road, Oxford, OX1 3PJ, UK; d Department of Neurosurgery, University of California, San Francisco, Eighth Floor, 400 Parnassus Ave, San Francisco, CA, 94143, United States

**Keywords:** Adaptive Deep Brain Stimulation, Parkinson’s disease, Sleep, Real-time neural control

## Abstract

**Background:**

Sleep dysfunction is disabling in people with Parkinson’s disease and is linked to worse motor and non-motor outcomes. Sleep-specific adaptive Deep Brain Stimulation has the potential to target pathophysiologies of sleep.

**Objective:**

Develop an adaptive Deep Brain Stimulation algorithm that modulates stimulation parameters in response to intracranially classified sleep stages.

**Methods:**

We performed at-home, multi-night intracranial electrocorticography and polysomnogram recordings to train personalized linear classifiers for discriminating the N3 NREM sleep stage. Classifiers were embedded into investigational Deep Brain Stimulators for N3 specific adaptive DBS.

**Results:**

We report high specificity of embedded, autonomous, intracranial electrocorticography N3 sleep stage classification across two participants and provide proof-of-principle of successful sleep stage specific adaptive Deep Brain Stimulation.

**Conclusion:**

Multi-night cortico-basal recordings and sleep specific adaptive Deep Brain Stimulation provide an experimental framework to investigate sleep pathophysiology and mechanistic interactions with stimulation, towards the development of therapeutic neurostimulation paradigms directly targeting sleep dysfunction.

## 1. Background

Sleep dysfunction is a unifying feature across many neurological and psychiatric disorders [1]. In people with Parkinson’s disease (PD), non-motor symptoms significantly decrease quality of life with up to 90% reporting significant sleep dysfunction across both rapid eye movement (REM) and non REM (NREM) stages [2-5]. In healthy individuals, NREM sleep is associated with an increase in cortical brain activity in low frequencies (0.5–4 Hz), named slow waves, which are believed to serve multiple functions related to metabolism, cognition and synaptic homeostasis [6]. In PD, reductions in slow waves are associated with faster disease progression [7]. Conventional, high frequency Deep Brain Stimulation (DBS) delivered to the Subthalamic Nucleus (STN) has been shown to partially improve sleep structure and NREM slow wave activity (1–4 Hz) in PD [8-11]. However, the variability of DBS on sleep overall, the impact of DBS on the globus pallidus interna (GPi) overnight, the mechanism by which DBS improves NREM sleep, and why REM sleep disturbances appear refractory to DBS is not well understood [12,13].

The observed improvements in sleep structure after DBS initiation are a fortuitous byproduct of stimulation optimized for daytime motor symptoms (inc. tremor, slowness & stiffness), rather than for overnight sleep physiology [7,8,14]. Furthermore, adaptive protocols for DBS in PD have primarily focused on beta activity, with minimal attention provided towards overnight slow wave activity [15]. Modulation of DBS stimulation parameters specifically adjusted to NREM and REM sleep stages plus neurophysiology and behavioral outcomes (e.g. RBD) would provide a critical tool to uncover the interaction between DBS and sleep neurophysiology. Identifying optimal parameters for individual sleep stages has the potential to advance new neuromodulatory therapies targeting sleep dysfunction in order to improve next day motor and non-motor symptoms, and potentially, through optimizing slow wave activity, to slow disease progression [7]. However, sleep physiology is multifaceted and exhibits dynamics across many frequency bands, conferring complexity beyond conventional beta-band focused adaptive DBS. Consequently, sensitive and specific modulation of stimulation parameters to individual sleep stages benefits from machine learning discrimination of sleep staging based on intracranial data.

We report a novel approach to sleep modulation in PD using a fully automated, adaptive DBS algorithm that adjusts stimulation amplitude according to sleep stage specific intracranial cortical biomarkers, demonstrated in two participants with PD. We target N3 sleep, as a proof-of-principle of sleep specific adaptive DBS, to investigate preliminary effects on slow wave activity and propose a pipeline that can be implemented fully remotely in patients’ homes to potentially target other sleep stages.

## 2. Material and methods

### 2.1. RC + S system

**Fig. 1.**
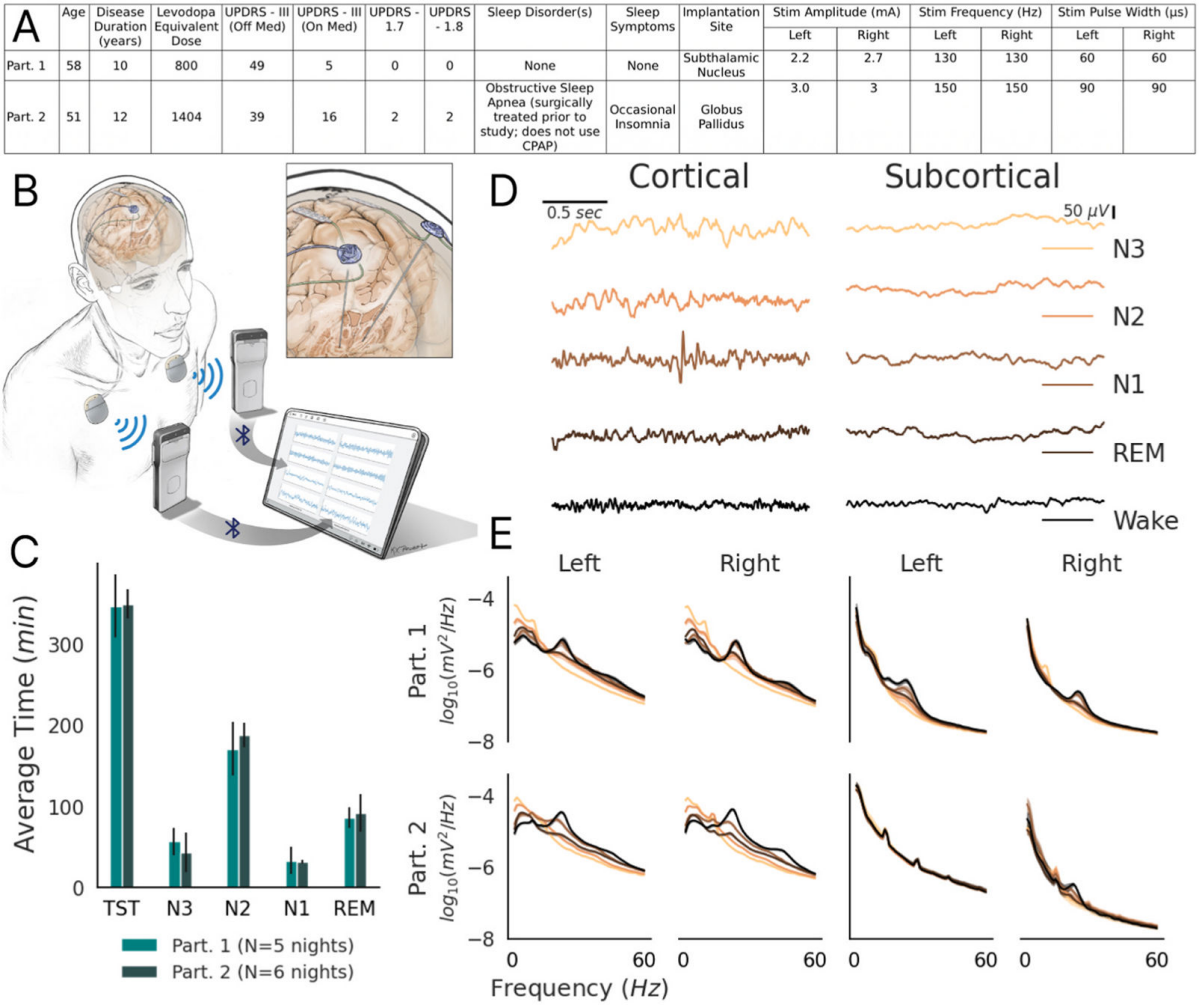
Participants’ (abbreviated Part.) recording set up and intracranial cortical Field Potentials (A) Clinical information for participants. UPDRS - Unified Parkinson’s Disease Rating Scale. (B) Schematic of RC + S system. The inset provides a close-up of the cortical and subcortical leads. Adapted from Gilron et al., 2021 [16]. (C) Average time spent in each sleep stage (TST = Total Sleep Time), per night, classified by Dreem2 headband polysomnogram data. Error bars indicate standard deviation across nights. (D) Representative traces of Field Potential time series in all sleep stages from Participant 1’s left device, with stimulation on: Left column - precentral gyrus; Right Column - Subthalamic Nucleus. Columns share color legend and scale bars. (E) Power spectral density plots of intracranial FPs, partitioned by sleep stage, during conventional continuous stimulation. Left Quadrant - precentral gyrus; Right Quadrant - subcortical region (STN for Participant 1; GP for Participant 2). Shaded error bars indicate standard error across nights; shares color legend with panel D.

This study was reviewed by our Institutional Review Board and registered on clinicaltrials.gov (NCT0358289; IDE G180097). We enrolled two participants diagnosed with idiopathic PD who provided written informed consent. Participants were implanted with bilateral electrodes in the STN (Participant 1) or Globus Pallidus (GP; Participant 2) nuclei. DBS targets were determined by the participant’s treating clinical team and both main targets were included in order to test the pipeline during the presence of stimulation at both STN and GPi. Implanted electrodes were connected to investigational sensing-enabled Summit RC + S (Medtronic) DBS implantable neurostimulators (INS), as part of a parent study investigating daytime closed-loop DBS for motor symptoms (Fig. 1B) [16]. Patients were programmed for conventional DBS by a movement disorder specialist, optimizing stimulation for daytime motor symptoms. Our electrode implementation consists of bilateral sensing and stimulation-capable quadripolar leads in the basal ganglia targets as well as bilateral quadripolar subdural electrocorticogram (ECoG) arrays spanning the precentral and postcentral gyri [16]. Field potential (FP) time series recordings were analyzed via time frequency decomposition through the Fast Fourier Transform (FFT) embedded within the INS. All data recordings and stimulation testing were performed remotely in patients’ homes. For safety, participants could manually switch from adaptive mode with personal programmers, if needed.

### 2.2. Polysomnogram and electrocorticography data collection

Participants streamed overnight subcortical and cortical (precentral gyrus) FPs concomitantly with extracranial electroencephalography (EEG) data from a portable polysomnogram (PSG, Dreem2 headband, Dreem Co., Paris, France [17]), while on clinically optimized chronic neurostimulation and dopaminergic medication. The Dreem2 headband provides scalp electroencephalography time series as well as automated sleep stage classification hypnograms, aligned with the American Academy of Sleep Medicine sleep scoring methods, but using an automated algorithm validated on healthy adult subjects [17,18]. The hypnogram of the participants’ sleep stages for a given night were time-synchronized via data timestamps, up to 1 s resolution, to the intracranial cortical and subcortical FP data during offline analysis.

### 2.3. Sleep stage classifier model development

We recorded five and six consecutive nights of PSG plus intracranial subcortical and cortical precentral gyrus neural data for Participants 1 and 2, respectively; totalling 30 h for Participant 1 and 36.3 h for Participant 2 (Fig. 1C). During deeper sleep stages of N2/N3, an attenuation of beta (12–30 Hz) and gamma power (30–60 Hz), with an increase in low frequency theta (5–10 Hz) and delta power (0.5–4.5 Hz) on cortical ECoG data was found, with less pronounced differences found subcortically (Fig. 1D-E). We therefore utilized cortical, rather than subcortical FP data for embedded RC + S sleep stage classification, in order to minimize stimulation related artifacts and to align with the cortical inputs from the automated hypnograms.

The RC + S INS has functionality to implement up to 2 linear discriminant classifiers, each using up to four spectral power bands as inputs. The INS’s embedded classifiers compute an inner product of a researcher-defined weight vector (w) with a vector of up to 4 feature inputs (x), and compares the result to a researcher-defined threshold (α):

$$\sum_{i=1}^{4}(w_{i})x_{i}=a$$

Above-threshold and below-threshold evaluations of the inner product lead to control policy changes of stimulation parameters, such as predefined increases or decreases in stimulation amplitude. We implemented a single classifier per INS. The feature inputs to the classifier were power data averaged over 60 FFT interval calculations of 1 s windows (250 samples) with 50% overlap and 100% Hann filter (equivalent to one 30 s sleep stage).

**Fig. 2.**
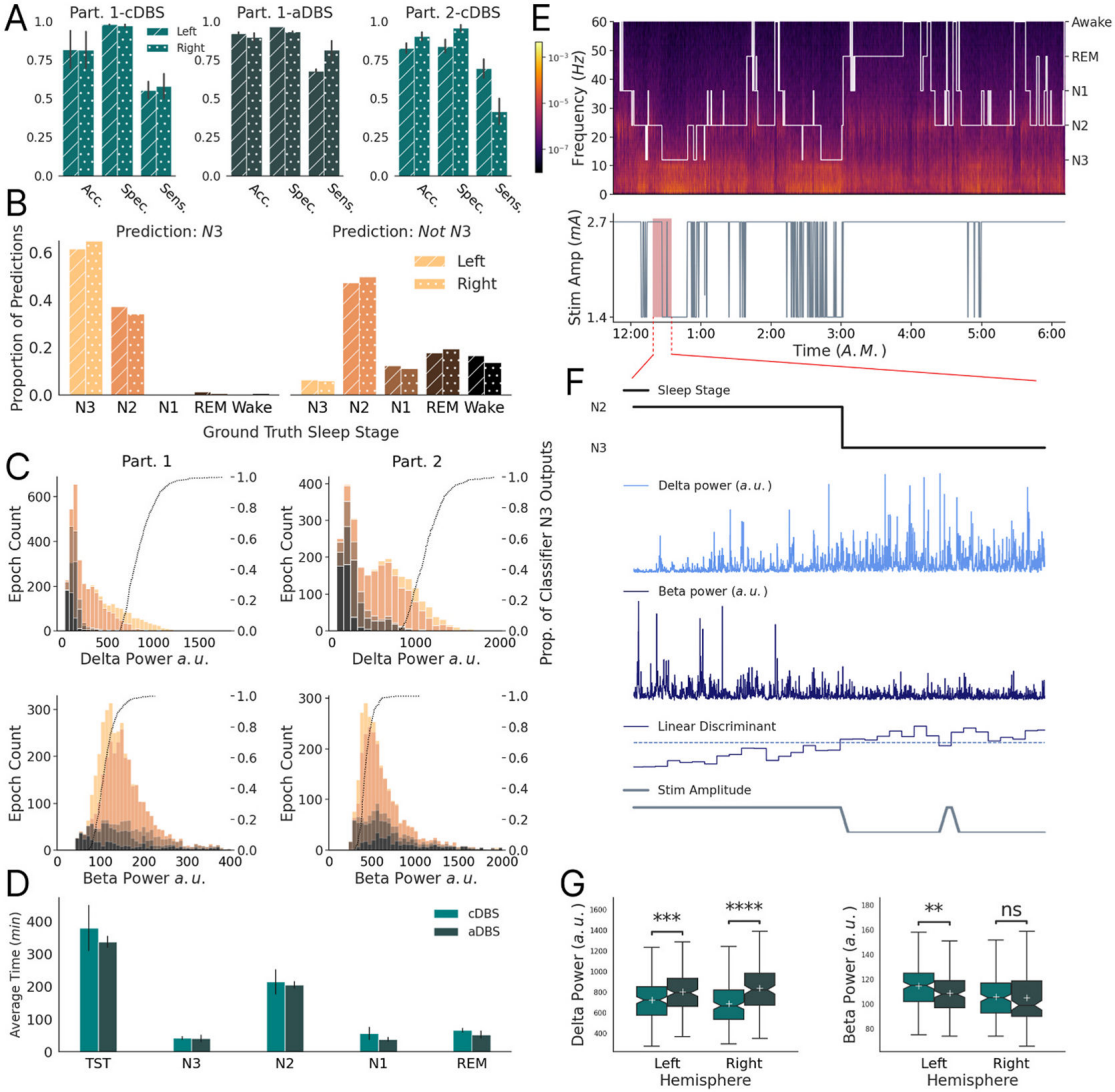
Sleep stage adaptive DBS classifier performance: (A) Classifier performance of the participant’s (abbreviated Part.) embedded N3 classifier during validation (cDBS: no adaptive stimulation change) and test (adaptive DBS - aDBS: stimulation changes, only Participant 1) phase nights. Error bars indicate standard deviation. (B) The proportional composition of Participant 1 and 2’s classifier outputs by ground-truth sleep stages during the validation and test nights. Utilizes the same color legend as Fig. 1D and E. Left plot depicts Dreem headband determined sleep stage composition of ‘N3’ embedded classifier outputs across all nights and participants. Solid bar and dotted bar correspond to left and right devices, respectively. For example, 35–40% of embedded N3 predictions in the left hemisphere device occurred during N2 sleep. Right column depicts the corresponding composition of embedded ‘Not N3’ classification. (C) Stacked histograms depicting number of 30 s sleep epochs with corresponding delta and beta power, color-partitioned by sleep stage (shares color legend with panel B). Dotted line represents cumulative distribution function of embedded left and right N3 classifications as a function of band power, illustrating the proportion of N3 predictions that occurred with sleep epoch band power less than or equal to the x-axis location. Participant 1 - left column; Participant 2 - right column. This demonstrates that classification sensitivity improves for progressively deeper N3 sleep. (D) Sleep metrics for Participant 1’s cDBS (validation) and aDBS (test) nights. Error bars indicate standard deviation. In particular, average N3 in cDBS is 42 min, while average N3 in aDBS is 41 min. (E) (Top) Dreem2 headband hypnogram superimposed on a spectrogram of precentral gyrus cortical FPs for one of Participant 1’s adaptive DBS test-nights. (Bottom) Stimulation amplitude as a function of time, sharing the same x-axis as the hypnogram. The stimulation amplitude was reduced (50%) during embedded classification of N3 (16.7 min span depicting a transition into embedded classification of N3 sleep). (F) Zoomed in depiction of the highlighted portion in panel D. Black line shows the ground-truth sleep stage. Below are the raw delta power (light blue), and raw beta power (dark blue) traces, as calculated by the embedded INS device, corresponding to the highlighted portion. Below depicts the corresponding Linear Discriminant embedded classifier output (blue) compared with the user-defined threshold (dashed). Grey line shows the resulting stimulation amplitude. All plots in F share the same x-axis. (G) Box plots of device-calculated band power, as described in Materials and Methods Section C, of all N3 epochs (cDBS → Left: n = 169; Right: n = 168 ∥ aDBS → Left: n = 162; Right: n = 134) across Participant 1’s cDBS and aDBS nights. Asterisks indicate significance of independent samples *t*-test (Delta → Left: t = −3.5, p < 1e-3; Right: t = −5.8, p ≪ 1e-3 ∥ Beta → Left: t = 2.7, p < 1e-2; Right: t = 0.2, p = 0.8).

We leveraged the canonical sleep bands (delta and beta) as feature inputs to train the offline Linear Discriminant Analysis (LDA) model (scikit-learn; Python) to classify N3 versus non-N3 sleep epochs (Fig. 2A) [19,20]. The entirety of the 5 and 6 nights for Participants 1 and 2, respectively, were used to train and develop the classifiers. We additionally tested inclusion of theta and gamma bands as input features for Participant 2, however this inclusion was not found to dramatically improve classification performance. LDA model weights were determined independently for each hemisphere, programmed into each INS’s embedded linear discriminant function, and validated *in vivo* over 2 consecutive nights (Fig. 2A-C). On validation nights the embedded devices performed real-time continuous N3 sleep stage classification with stimulation amplitude kept continuous (cDBS). For Participant 1, two further test nights were run in which positive N3 classification resulted in a 50% reduction in stimulation amplitude for the subsequent 30 s epoch (aDBS; Fig. 2D-E). Stimulation amplitude reduction during N3 sleep was chosen for the safety and tolerance of the participant.

## 3. Results

We demonstrate high specificity (0.94 ± 1.4e-2) for classification of N3 sleep using intracranial cortical embedded neural classifiers, and well above chance sensitivity (0.62 ± 4e-2) across subjects and hemispheres (Fig. 2A). Most false positives corresponded to misclassification of N2 sleep, which has an overlapping spectral profile to N3 (Fig. 2B-C). N3 epochs with ‘deeper’ profiles (i.e. elevated intracranial cortical delta power and reduced beta power), further increased the sensitivity of embedded N3 classification (Fig. 2C). In the two aDBS nights, there was successful reduction of stimulation amplitude for 67% and 83% of left and right recorded N3, respectively; with incorrect stimulation modulation in only 3% and 6% of left and right non-N3 (Fig. 2E-F). Classifier performance was not affected by any potential sensing contamination from stimulation adjustments (Fig. 2A) [21]. No differences in time per sleep stage between the cDBS and aDBS nights were observed (Fig. 2D). However, there was an increase of mean delta power on the left (11%) and right (22%) during N3 epochs for the aDBS nights when stimulation was reduced (Left: t(331) = −3.5, p < 1e-3; Right: t(302) = −5.8, p ≪ 1e-3; Fig. 2G).

## 4. Discussion

We demonstrate proof-of-principle of intracranially controlled, embedded, adaptive DBS targeted to N3 NREM sleep in two participants with PD. Our approach demonstrated high specificity for stage N3 sleep and sleep stage adaptive DBS was well tolerated, with stimulation changes causing no detectable adverse effects. High specificity (low false positives) is favorable from a clinical perspective, as it reduces unnecessary changes from therapeutic stimulation in untargeted sleep stages. Sensitivity can likely be further improved through the use of subject specific features inputs as opposed to canonical power bands, and tuned to a desired mark by modulation of the LDA threshold. Although a 50% reduction in stimulation amplitude during embedded N3 classification was primarily chosen for safety reasons, the adaptive stimulation paradigm also provided evidence for an increase in slow wave activity. We propose that slow waves are likely suppressed by both intrinsic pathophysiological neural rhythms such as beta (13–30 Hz) oscillations as well as excessively high DBS amplitudes [22]. As beta is itself also suppressed by DBS, this may result in a subject-specific inverted U shaped curve relating stimulation amplitude to NREM slow wave amplitude. Additionally, there are likely other complex, non-linear interactions between DBS sub-harmonics and underlying slow wave entrainment that may allow for an increase in endogenous slow wave activity at optimal DBS amplitudes [23]. Slow wave activity has been linked to PD disease progression and therefore, if confirmed over larger numbers of nights and subjects, slow wave optimization through adaptive DBS may represent a promising novel potential therapeutic approach in PD [7,22]. This is a step towards implementing stimulation protocols to investigate the optimal overnight stimulation amplitude during N3 to support maximal slow wave activity, which we predict will be subject specific. Taken further, tailored DBS delivery during alternative individual sleep stages may help recover normal sleep physiology and metrics in people with PD, addressing a primary non-motor symptom of Parkinson’s Disease.

Limitations to this proof-of-principle study include ground-truth sleep stage labeling obtained through a portable polysomnogram and automated sleep-scoring algorithm [12]. However, simultaneous intracranial recording demonstrated expected, canonical, ECoG power band changes in different sleep stages classified by the Dreem2 band, notably an increase in delta power during N3 sleep, supporting dissociation of underlying sleep stages in our patient population. Additionally, our portable remote setup supports multi-night recordings in natural settings for improved sleep quality and classification model training, compared to single night sleep laboratory PSG. We also report a small sample size, and do not leverage subcortical data for N3 classification nor incorporate subjective measures of sleep quality. Nonetheless, multi-night, at-home recordings support within subject, individualized, sleep classification models and the proposed methods have flexibility to accommodate expanded participant cohorts, different stimulation targets and inclusion of auxiliary intracranial data streams. Additionally, subjective metrics of sleep quality can be used as an outcome measure for a more complete assessment of sleep aDBS paradigms.

Translation of the proposed pipeline for patient care might be accelerated if sleep stages could be classified from subcortical electrodes. Complementary studies have shown STN and GPi field potentials display distinct NREM vs REM physiologies in people with PD, and resulting sleep stages can be discriminated in the absence of stimulation [24-26]. Therefore, the proposed approach could be adjusted to include subcortical field potentials as feature inputs to the personalized linear classifier, although local stimulation related artifacts and signal distortions might reduce classification accuracy.

Personalized sleep stage adaptive DBS provides a technique to investigate sleep neurophysiology in PD. Additionally, this approach could be leveraged towards adaptive therapies that target sleep symptoms and potentially impact next day motor and non-motor functioning in PD [27-29].
